# Selections and Abstracts

**Published:** 1899-10

**Authors:** 


### SELECTIONS AND ABSTRACTS.

### Diagnosis of Abdominal Contusions with Hollow Visceral Lesion.*

Few emergencies are more embarrassing than when standing by a patient who has had an abdominal contusion we try to ascertain, by the symptoms manifested, the existence or absence of an intra-peritoneal lesion.

A ruptured solid or hollow viscus means death, either from hemorrhage or septic peritonitis. Such injuries, it is claimed, are even more fatal than gunshot wounds of the abdomen, and certainly not nearly so many cases have been diagnosed and operated upon. The key to the successful treatment, we all know, lies in an early diagnosis. Every hour of delay greatly increases the risk, and the successful surgeon will be the one who gets into the abdomen early and out of it just as quickly as is consistent with thorough work. Ideal surgery is preventive in its tendencies. An early operation prevents profuse hemorrhage in solid visceral wounds as well as general septic or suppurative peritonitis—the inevitable sequence of hollow visceral lesions. An early operation limits the operative intervention needed, and therefore is conservative in that it is accomplished prior to the depression of acute anemia and the ptomain intoxication of acute sepsis.

It is a waste of words to advocate prompt intervention when we know we have a hollow visceral wound to treat, nor need we dwell upon the technique. What we most need in this, as in so many departments of surgery, is not an improved technique, but better means of diagnosis.

An early operation entails an early diagnosis, but unfortunately in few cases only is the early clinical picture clearly portrayed by classical symptoms; *per contra*, in many instances the symptoms are inadequate and certainly misleading until the case has passed

*Read before the Southern Association of Railway Surgeons, at Richmond, Va., May 29, 1899.

from the safe operable to the desperate stage. Our interest then must necessarily be largely focused in the diagnosis and obviously upon the early symptoms. My own limited experience and a study of the recorded observations of others impresses the conviction that the high rate of mortality must continue an opprobrium to modern surgery until we devise means by which we can diagnose such lesions in their incipiency. It goes without saying that a blind man can make the diagnosis when the tripod—peritonitis, tympanitis, and vomiting, the furies of so many intra-abdominal traumas and phlegmons, have reach their acme.

Can an early diagnosis be made? Must it, with our present knowledge, remain largely a matter of conjecture? Within what restrictions is a confirmatory incision warranted? These are momentous questions with which most of us have been, and with which any of us may be confronted. Can an early diagnosis be made by the symptoms usually presented? Is there any uniformity in the symptoms manifested? As a rule we cannot make an early diagnosis, except by an exploratory celiotomy. We may suspect and dread a lesion, may be keenly alive to the danger of procrastination, and yet, with few exceptions, there is an element of doubt as to the diagnosis; and inasmuch as we cannot be emphatic, it is wrong to be dogmatic. A trinity of sequences common to many intra-abdominal lesions is shock, hemorrhage and sepsis, and the first mentioned as an early symptom merits more than a passing notice.

*Shock.*—Unfortunately the presence or absence of shock is no guide as to the existence of either hollow or solid visceral lesion. It is too well known that a trifling blow upon the abdomen may be attended by a profound shock, while extensive or multiple visceral lesions from either penetrating or contused wounds of the abdomen may exist with no appreciable shock. While the presence or absence of shock is no guide, its behavior when present may shed some light. Shock incident to a trauma, limited to the abdominal wall, or even in the case of a penetrating wound without visceral lesion, should be immediate in its onset, should reach its acme at once and disappear under appropriate treatment in a short time. Prolonged shock points to a visceral lesion. Shock followed by reaction and recurring shock points strongly to visceral lesion, the recurring shock being due to intraperitoneal hemorrhage or the escape of the contents of a hollow viscus into the peritoneal cavity. Progressive shock succeeding reaction points to solid visceral lesion, *i. e.*, of the liver, kidney, spleen mesentery or omentum. Shock reaction and renewed shock in a few hours points to shock of hemorrhage. Shock reaction and renewed shock in 12 to 24 hours points to the advent of septic peritonitis and the shock of sepsis. Shock of trauma may exceptionally be prolonged and be merged into the shock of hemorrhage or sepsis, and it is equally true that the shock of extravasation into the peritoneal cavity prior to the shock of sepsis may be prolonged. The shock of hemorrhage is slow in its onset, or rapidly progressive in proportion to the size and number of the bleeding vessels. It is claimed by Greig Smith that the symptoms of simple contusion are more acute at first than those of rupture, and that profound and immediate shock is probably more frequent in cases of simple contusion than in cases of genuine rupture. A majority of clinicians agree that shock *per se* is no guide, but I think shock may be presented in such a way as to make its manifestations significant.

*Abdominal distention*, incident to septic peritonitis and intestinal paresis, is a common late manifestation in both hollow and solid viceral lesion, but it is an open question if it uniformly exists to an appreciable extent in the incipiency of even hollow visceral wounds. Invariably I have looked for distention coming on rapidly, which would point to an escape of gas and fluids into the peritoneal sac, and, without an exception, I have failed to appreciate their presence.

A case seen a few days ago is an apt illustration: A young man had been sick for several weeks with typical enteric fever. At 10 o'clock A. M. he had a bowel movement and immediately suffered violent abdominal pain, and vomited once. When I saw him at 1 o'clock his pulse was 130, temperature 105°, abdomen flat but rigid, and there was no movement of gas in his intestines appreciable either with the ear directly applied or with the aid of the phonendoscope. The sudden onset of pain, vomiting, pulse increase, bad facies, rigid abdomen and abolished peristalsis warranted the diagnosis of perforation. The area of hepatic dullness was carefully examined, and no appreciable diminution could be ascertained. While the pulse was steadily going up, his temperature did not come down, as might have been expected from the shock of effusion into the peritoneal cavity. On making an abdominal section two hours later (five hours after the perforation occurred) the peritoneal cavity was found to contain serum and fluid fecal matter, but no gas escaped when the peritoneal sac was incised. Two perforations as large as a silver dime were found in the ileum within twelve or fourteen inches of the ileocecal junction. Doubtless the temperature would have declined in eight or ten hours more from the shock of sepsis, and in that time abdominal distention from septic peritonitis would have occurred. Theoretically, we should expect in gastro-intestinal perforations an escape of gas into the peritoneal cavity, and its distention and the further effect of obliterating hepatic dullness. Much stress has been placed upon the value of this symptom. My experience impresses the belief that it is an evidence often notably absent. I have usually found the intestines collapsed only immediately around the rupture. Attention has been called to the fact that adhesions between the liver and parietal peritoneum will prevent this symptom being manifested, and also the fact that a distended transverse colon may be pushed up above the convex surface of the liver, and thus give resonance over the hepatic area.

*Abolished Peristalsis.*—My interest in this important symptom was much increased by the emphasis given by Dr. Murphy, of Chicago, several years ago. "If," he says, "the patient has perforation of the bowel, he has obliteration of peristalsis, which is one of the most positive signs of intestinal perforation." My experience confirms the opinion that this symptom is notably valuable, in that it is one of the earliest manifestations, being appreciable at a time when reliable symptoms are but few, if any, and at a time when it is so essential to make a diagnosis. Within the past few months I have had the opportunity of valuing this symptom in one case of bullet perforation, in two of typhoid perforation, and in one of duodenal perforation. In each instance the case was seen within a few hours after the occurrence of the bowel lesion. In all this symptom was particularly studied by myself and colleagues, and in each case an obliterated peristalsis did much to confirm the diagnosis of perforation and to warrant operative intervention. I can but regard this symptom as furnishing the most conclusive preseptic evidence of perforation we have at our command. In a case recently seen, one of bullet wound of the abdomen with eighteen perforations, nine intestinal and nine mesentric, this symptom was marked. On making a section we were surprised to find so little extravasation into the peritoneal sac. The size and number of the lesions do not seem to regulate the amount of effusion. It is probable that the obliteration of peristalsis is due to nerve trauma, and is in one sense conservative, as its obliteration lessens extravasation of the intestinal contents.

*Muscular Rigidity.*—We have been led to regard muscular rigidity as a symptom of perforation as being second only in importance to an abolished peristalsis. I have found it uniformly present when extravasation occurs, and as an early symptom, it is one of especial value. I have uniformly looked for and have, as far as I can recall, always found it present, in a marked degree, in cases of perforating gastro-intestinal lesions. In atypical appendicial infections I have seen it absent, either when the local inflammatory trouble was very slight, or when with a gangrenous appendix, or general septic peritonitis, the shock of ptomain infection was very marked, but with every sudden invasion of the peritoneal cavity it has been a conspicuous symptom. Many writers impress the value of this symptom; not a few regard it as almost infallible, and with these last my limited experience prompts me to concur.

*Pain.*—I do not think the intensity of the pain, its locality or its type, is a guide as to the presence or absence of an intraperitoneal lesion. We have all seen penetrating and non-penetrating abdominal wounds with visceral lesions in which the pain was slight, and certainly not indicative of the fatal intra-abdominal lesion. On the other hand, a slight blow upon the abdomen may induce sufficient pain without visceral lesion to induce profound shock. Notably, in penetrating bullet wounds able clinicians have impressed the common occurrence of acute pain, and some have even defined its type. Other equally close observers have but little confidence in pain as a guide, and my own experience sustains this conclusion. I am sure I can recall more than one case in which there was profound shock and so little pain that it was hard to impress the patient with the idea that the injury was of a serious nature. As pain may be pronounced when there is no visceral lesion, and as conspicuously absent when there is serious lesion, it cannot be regarded as in any sense diagnostic.

*Vomiting.*—I do not think the occurrence of vomiting or its absence affords reliable information. A case recently seen is illustrative: A child was kicked over the right iliac region by a playmate. He suffered pain for several days and vomited for ten or twelve hours. I was asked to see the case a week later, because of a suspicious suprapubicswelling, which proved to be an over-distended bladder. In a case of duodenal perforation there was no vomiting; in two cases of typhoid perforation vomiting occurred in each but once. In a case of bullet perforation of the stomach, the patient vomited once (no blood), while in another case of multiple bullet perforation of the bowels there was no vomiting. A simple trauma of the abdominal wall may induce shock, nausea and vomiting, and this is equally true of an intraperitoneal trauma of minor import. Intraperitoneal trauma with lesion of solid or hollow viscera will probably, as a rule, cause nausea and vomiting of the contents of the stomach, but this is very unlike the late gulping so common in septic peritonitis. As a late symptom of serious lesion, vomiting is common; as an early index, it is not significant. Vomiting of blood is suggestive of stomach lesion, but we have always held that a complete rent of the anterior wall of the stomach will result in the escape of the blood into the peritoneal cavity, and that in a trauma involving the mucosa and muscularis alone we will be more likely to have vomiting of blood.

*Facies.*—Inasmuch as slight injuries may be associated with acute pain and profound shock, while mortal injuries may be painless, and be attended with no shock, we cannot expect the face of the patient to afford a reliable index of the nature of the lesion. I have seen a man with multiple visceral lesions with an untroubled countenance, while the shock of a simple trauma may be markedly impressed upon the face. The face of a septic peritonitis is, as a rule, easily read, but as a guide in the early stages of an intra-abdominal trauma, I think the facial expression is by no means an unvarying guide.

*Pulse.*—My experience impresses the opinion that a rapid pulse is always a significant late symptom, and notably is this true if there is a falling temperature and increasing pulse-rate. A rapid pulse in the incipiency may point to physical, traumatic or hemorrhagic shock. Temperament influences the pulse to such an extent that it is no easy task, in all instances, to tell the neurotic pulse from that due to shock, hemorrhage or sepsis.

*Senn's Hydrogen Gas or Insufflation of Filtered Air under Pressure.*—Theoretically distention of the stomach, intestines, or bladder, by Dr. Senn's method would seem to be a test of value, and one which is applicable in the early stages of the injury, and far more confirmatory at that time than subsequent to the development of septic peritonitis and the distention incident to septic paresis. As I have never used this diagnostic means so emphatically approved by Dr. Senn I cannot speak experientially of its value. I do not think, however, it has received the attention from surgeons it merits, and I certainly think the trouble incident to its use is no excuse for the failure to give it a trial if it is as really valuable as it is claimed. Of course, it is only of use in excluding a lesion of one of the hollow viscera.

*Percussion of the Abdomen.*—It has been held that a gastro-intestinal lesion would result in extravasation into the peritoneal sac; that this extravasation would accumulate at dependent points, and its presence be elicited by percussion. I have never met with a case of hollow visceral wound in which anything like a sufficient extravasation took place to give the slightest evidence of its presence through percussion. In the few cases of profuse intraperitoneal hemorrhage I have seen the accumulation has usually been in the pelvis, and not in right and left lumbar region. I do not think information of value can be obtained by percussion.

Able clinicians concur in thinking the symptoms manifested are inadequate in many instances to enable one to differentiate between the abdominal contusion with, and one without, visceral lesion. So fatal is delayed operative intervention, certainly in hollow visceral wounds, that many urge an exploratory celiotomy in the absence of confirmatory symptoms.

Our interest is largely focused in the diagnosis, and especially in the early diagnosis, and I trust I am not altogether wrong in my valuation of the evidence afforded by abolished peristalsis, muscular rigidity, and the behavior of shock when it occurs.—*Taylor, Bi-Monthly Bulletin, Richmond, Va.*

---

### LA GRIPPE: ITS MANIFESTATIONS, COMPLICATIONS AND TREATMENT.

BY W. W. GRUBE, A.M., M.D., TOLEDO, O.

Professor of Physiology and Clinical Medicine, Toledo Medical College, Toledo, Ohio.

(Abstract from the *Journal of the American Medical Association*, March 25, 1899.)

Professor Grube sees no reason why the intelligent observer need err in his diagnosis of la grippe; he believes that the intensity of the catarrhal symptoms, the great prostration, and tardy convalescence form a typic clinical picture. Though the catarrhal symptoms are usually limited to the respiratory mucous membrane, they are not always so, and in the writer's experience the invasion of the mucous membrane of the digestive tract has been quite frequent. Not alone mucous membrane, but a part or all of the cerebro-spinal axis has been invaded.

In many cases the so-called complications are simply an extension and aggravation of the catarrhal or inflammatory condition; thus an extension of the usual inflammatory condition of the throat through the Eustachian tube produces middle-ear complications; the bronchitis, too, may extend and become capillary, or even a pneumonitis may result. So we believe that in the so-called abdominal form with severe gastro-enteric catarrh, it may extend by *contiguity* and inaugurate a general peritonitis. Upon this theory alone can we explain the supervention of a severe general peritonitis in a case under our care, now happily terminating in convalescence.

The patient was a girl of eleven years, who had never been seriously ill before. Twenty-four hours after the illness began, she had, besides the usual alarming symptoms of la grippe, a high temperature, wild delirium, constant emesis, frequent and copious discharge of feces and urine. The appropriate remedies were prescribed, the vomiting ceased and she rested; but on the third or fourth day she developed symptoms of peritonitis, abdominal pain, hardness and some tympanites, etc. Calomel was prescribed, twenty grains divided into four powders, one every three hours; also the usual turpentine stupes, morphia to quiet pain, etc. The next day, finding no improvement, but rather aggravated symptoms, green vomit, bowels not moved—a very gloomy prognosis was given, and at the family's request a consulting physician was called, who concurred in diagnosis and prognosis, and had nothing more to suggest. On the writer's return in the evening, however, he decided in view of the great mortality of these cases by the routine treatment, to try the local application of a mustard poultice; also, for their germicidal, antiseptic and healing qualities, he gave internally Hydrozone diluted, in frequent doses, alternating with doses of Glycozone. In twenty-four hours there was slight improvement. In forty-eight hours the patient was decidedly better. Improvement continued, and the girl was so well, February 21st, that she was dismissed as cured.

Perhaps the most common complication in children is the middle-ear inflammation caused by extension of the pharyngeal catarrh up the Eustachian tube into the tympanum. In the case of a child six months old, recently under our care, we had a middle-ear complication, in which the pain was controlled by the usual methods and by the instillation into the aural canal of a few drops of cocaine solution. After suppuration occurred, however, the canal was cleansed by Hydrozone solution (warm), and a piece of absorbent cotton saturated with Glycozone used as a dressing by inserting it into the canal. As the ear complications sometimes prove very serious, it is gratifying to know that in the above remedies we have a safe, speedy and effectual method of cure. We believe also that, if these cases were seen early, by proper treatment the extension and consequent complications might be prevented. In a little girl with severe tonsillitis and pharyngitis we are now spraying the throat with diluted Hydrozone and applying

Glycozone with such marked benefit that on this, the third day of treatment, she is almost well.

In concluding Professor Grube states: "I cannot refrain from referring to the case of a prominent city official who had an unusually severe attack of la grippe. All the structures of the nasal cavities were involved in a severe acute catarrh, which progressed to the stage of suppuration. Enormous quantities of pus were secreted, and the location and intensity of the pain led us to fear involvement of the antrum. However, the free use of Hydrozone solution by spraying, and the application of Glycozone soon cleared up the cavity, and in a few days complete cure resulted."

---

### SURGICAL HINTS.

All hypodermic injections may be rendered less painful, and be more readily absorbed if the active substance is dissolved in saline solution instead of plain water.

In nursing women, every inflammation of the breast and nipple must be considered as having a bacterial origin, and should be treated like any other local infectious process.

In alcoholic coma always investigate the bladder. It is apt to be very full. If there is no stricture the urine would drain itself out after a while, but if prostatic or other stricture should exist a rupture of the bladder might take place.

In administering chloroform to patients who have to be placed upon the side, as in some obstetrical operations, etc., place them on the right side if possible, as the heart's action is much better under chloroform in that position, than it is when the left chest is compressed against the table or bed.

In men, the intense scalding during urination in acute gonorrhea may be relieved by urinating with the penis immersed in a vessel containing hot water. Women with gonorrheal urethritis may similarly be relieved by directing them to urinate while taking a copious hot douche, or while sitting in a warm sitz bath.

After having succeeded in passing a catheter through a stricture, after some trouble, it is better to wait for some hours before withdrawing it. If you do not you may have just as much trouble in introducing another, whereas a catheter left *in situ* for a day or so will dilate the canal enough to allow you to pass the constriction quite easily.

In ankylosis resulting from disease still existing, passive motion is harmful. The only manipulation allowable in such cases is for the purpose of placing the limb, if possible, in the most useful position. In deforming arthritis, for instance, knees should be straightened out and elbows bent to a rather acute angle, under anesthesia. Then use rest, with splints and ice bags to prevent inflammation.

In women climacteric hemorrhages sometimes occur as the result of vasomotor disturbances or of arterial sclerosis. It sometimes happens that several such hemorrhages take place prior to the final establishment of the menopause. Women at this period always attribute such an occurrence to the change of life, but the surgeon must invariably examine the patient on account of the strong chances of cancerous trouble.—*International Journal of Surgery.*

---

### THE CURE WITH SODIUM BROMIDE OF HABITUATION TO MORPHINE, CHLORAL AND COCAINE.

McLeod (*British Medical Journal,* April 15, 1899) recommends the following mode of procedure: Having taken the weight of the patient and ascertained the absence of contraindications, sodium bromide is administered in doses of two drachms in solution every two hours for the first two days and of one drachm on the third day. None is given after bedtime on the third day. Three ounces of the drug in all will probably suffice, but more may be administered to induce deep sleep that may continue for five or six days and nights, during which time milk alone should be given. Night and morning the patient should be placed on the commode for evacuation of bowels and bladder, or oftener if there is any sign of soiling the linen. Solid food should be given as soon as it can be taken, and when locomotion is recovered exercise should be encouraged. Morphine or cocaine may be continued the first day in the habitual dose, halved on the second day, and withdrawn or given in small dose on the third day. Chloral may be cut off at once if there is good sleep on the first night. A case may appear well in three weeks, but at least three more weeks should be allowed for convalescence.—*Medical Record.*

---

### ABSENCE OF SELF-RESTRAINING POWER IN A MAN CONVICTED OF PUBLIC INDECENCY.

M. Bérillon (*Indépendence Médicale*) recently presented to the French Society of Hypnology and Psychology a patient who had been convicted of indecent behavior, and pointed out that he was the subject of urethral strictures, one situated in the bulbar and one in the membranous portion of the urethra; to the latter one spasm was superadded. These lesions, M. Bérillon considered, were the explanation of the reflex nervous troubles which resulted in the acts for which he was prosecuted. At certain times, under the influence of urethral irritation, the patient fell into a state of genital erethism, inducing the automatic accomplishment of indecent acts. Under the influence of genital irritation the spinal centers were relieved from the controlling psychomotor influence of the cerebrum; the lower centers became independent, functioning irresistibly. The man was deprived of controlling power, whence he ought not, in the author's opinion, to be held responsible for irresistible reflex impulses. The treatment required to be directed both to remove the cause of the impulses and to increase the power of self-control.—*Ex.*

---

### UREA.

For the estimation of urea, the following from Bartley's chemistry is recommended: "A graduated tube is filled to the fifth division with a 20 per cent. aqueous solution of potassic bromide. Chlorinated soda solution (Squibb) is then added to the fifteenth or twentieth division. Pure water to the depth of one inch is now floated upon the contents of the tube. It is most easily deposited there with the aid of a pipette. One cc. of urine is then floated upon the water, taking care that the liquids do not mix. The open end of the tube is now quickly closed, by pressing the thumb firmly upon it. The contents are mixed by gently shaking. When the effervescence has ceased, the reading is taken at the top of the liquid column, while the tube is held inverted. The end of the tube, still closed by the thumb, is now submerged in a large jar of water. The thumb is then removed, and the tube raised or lowered till the surface of the liquid in the tube is at the same level with that in the jar, and the reading is taken again. The difference between the two readings indicates the number of grains of urea in a fluid ounce of the urine. This number, multiplied by the number of fluid ounces of urine voided in twenty-four hours, gives us the total quantity of urea excreted during the day. The average daily amount is about 500 grains, but it is subject to considerable variation within the normal limits.—*The Practice of Obstetrics, Jewett.*

---

### TWO CASES OF TETANUS SUCCESSFULLY TREATED WITH ANTITOXIN.

Patteson (*The Dublin Journal of Medical Science*) reports two cases of tetanus in which the symptoms became very alarming and where the use of antitoxin seemed to have been the cause of the ultimate recovery. The author says that he is aware that the cases are open to the obvious criticism that they belong to the type of tetanus which would recover if left to the *vis medicatrix naturæ* alone. But he answers that he has seen cases with no more pronounced symptoms rapidly run to a fatal termination, and one is not justified in standing idly by while a remedy full of promise lies ready at hand.

Whether the future will justify the hope based on serum therapeutics can only be determined by a careful record of cases, and all should be reported, and in this direction lies at present our hope of combating some of the most terrible infective ills that humanity can ever suffer from.—*New England Medical Journal.*

---

### A NEW OPERATION FOR THE RADICAL CURE OF HYDROCELE OF THE TUNICA VAGINALIS.

Dr. J. J. Pratt (*The Indian Medical Gazette*) describes an operation which he believes to be specially suitable to hydroceles of moderate dimensions. In the enormous hydroceles he thinks it would be best to perform a modified excision, removing the anterior part of the sac before everting. He performs the operation as follows: After careful shaving and thorough washing and cleansing of the parts, the scrotum is made tense by being firmly grasped in one hand, an incision is made along the whole length of its long axis, the tunica exposed, and the testicle almost entirely withdrawn from the scrotum; then, the tunica having been punctured with the knife, the puncture is enlarged with the scissors to a sufficent extent to allow of the testicle being drawn out through the opening. This having been done, the parietal tunica is turned inside out, and the opposite edges of the incision in the sac are united behind the epididymis by a single catgut suture. The cavity of the tunica thus ceases to exist, and the testicle and epididymis are covered almost completely by one continuous layer of serous membrane. The skin incision is closed with a continuous suture, and the operation completed.—*Medical Record.*

---

### SCHLEICH'S GENERAL ANESTHESIA NOT A SUCCESS.

His personal experience in a large number of cases leads H. Rodman (*Medical Record*) to look unfavorably upon this new anesthesia mixture, although the favorable reports which followed its introduction into this country had led to the hope that it would obviate some of the unpleasantness and dangers attending the administration of ether or chloroform. He has not found it less free from danger than either of the other anesthetics; has seen dangerous cyanosis and syncope. Has found the same harmful effects upon the lungs and kidneys and proved it to be a respiratory and cardiac depressant. Its only advantage is that it is pleasanter to inhale. It is not safe in heart lesions, for it was shown that a sound heart can be so depressed by its action as to greatly endanger the patient's life. These poor results have caused the withdrawal of the mixture almost entirely from the hospital where the results were observed.—*International Medical Magazine.*

### THE CHANCRE OF SYPHILIS.

The diagnosis of syphilis is a serious and important matter, says Dr. Thomas in the "*International Medical Magazine*," and he takes up the diagnostic points in regard to the chancre:

(1) The initial lesion, (2) adenopathy, (3) the eruption, with concomitant symptoms of each step. Clinical history very important. The initial lesion is the chancre, the growth slow, ten days to three weeks before appreciable; hardly ever later. Of all diseases the development of syphilis is most uniform.

Some solution of continuity of the body envelope gives entrance to the poison. Exceptionally we have more than one chancre. The abrasion sometimes heals over the poison, an indurated papule, which later breaks down and ulcerates. We have virtually but two varieties; eroded and non-eroded.

Chancre may be insignificant. In the female it is hard to get a good demonstration of the chancre. It may resemble mucous patch, moist papule or abrasion. The position of the enlarged glands may aid in determining the situation of the lesion. The author states that the eruption almost always appears about sixty days from the infection.

Local treatment for chancre is cleanliness; cauterization worse than useless; excision should not be done; constitutional treatment the main thing.—*Charlotte Medical Journal*, August, 1899.

---

### CONSTIPATION CONSIDERED FROM THE STANDPOINT OF THE PROCTOLOGIST

was the title of a paper read at the meeting of the American Society of Proctologists, by Dr. A. B. Cooke of Nashville. He defined constipation as a diseased condition of the alimentary canal, characterized by a modification of function which resulted in the pathological retention of fecal matter. He mentioned among the causes those springing from the violation of hygienic laws; defective innervation, expressed either in atonicity of the muscular coat of the intestine or in decreased secretion; sluggishness of bowel function; the habitual use of purgative medicines; mechanical obstruction; and painful affections of the anus. The relations between constipation and diseases of the rectum, he said, were intimate and noteworthy in that either might be the cause or effect, with reference to the other. Rectal reflexes came in for a fair share of consideration. It was the author's conviction that in a large proportion of cases constipation either originated in or was maintained by conditions situated in the distal ten inches of the intestinal tract. If this was true, he said, the notorious inadequacy of ordinary treatment was at once accounted for, and the duty of the proctologist in the premises became obvious.—*New York Medical Journal.*

---

### TYPHOID GERMS TRANSMITTED BY THE ATMOSPHERE.

According to the "*Journal d'Hygiene,*" recent experiments have proved that the typhoid germ can be transmitted by the atmosphere. The germ preserves its vitality a considerable time, not only in damp surroundings, but in others apparently dry, such as linen, wood, earth, dirt and refuse. By the process of desiccation the greater part of the bacilli perish. A few, however, escape, and these may become a source of danger, not only by being transmitted by the surrounding air, but also because imperceptible fragments containing them, by being brought into contact with the buccal mucous membrane, from fingers, food, etc., produce infection.

Hare believes that the cold-bath treatment of typhoid fever has reduced the case-mortality by 50 per cent., that nearly all the distressing symptoms connected with the pyrexial state, and many of those dependent upon the intestinal lesions are alleviated to an extent not attainable by any other therapeutic measure, that nutrition is maintained and convalescence greatly accelerated, and that in cases that terminate fatally life is prolonged on the average by several days. He thinks the application of this treatment should be routine. The selection of suitable cases is out of the question. —*Southern California Practitioner*, August, 1899.

---

### GENERAL SEPTIC PERITONITIS.

A. J. McCosh states that the use of sulphate of magnesia—one to two ounces saturated in aqueous solution injected into the small intestine during laparotomy for general septic peritonitis—is a valuable addition to the usual method he has employed. The steps of his technique are as follows:

1. Chloroform narcosis.
2. A free incision—five to six inches.
3. Usually evisceration.
4. Removal of the cause of peritonitis.
5. Irrigation with salt solution ot 110° to 112° F.
5. Sulphate of magnesia solution injected into the small intestine.
7. Drainage.
8. Partial closure of wound.

As to after treatment, he gives ten grains of calomel supplemented by rectal stimulation.—*Annals of Surgery.*

### HOT WATER DRINKING.

There are four classes of persons who should not drink large quantities of hot water. These are as follows:

1. People who have irritability of the heart. Hot water will cause palpitation of the heart in such cases.
2. Persons with dilated stomachs.
3. Persons afflicted with "sour stomach."
4. Persons who have soreness of the stomach, or pain induced by light pressure.

These rules are not for those who take hot water simply to relieve thirst better than cold water, and for that purpose is not to be condemned. But hot water is an excitant, and in cases in which irritation of the stomach exists, should be avoided.—*Indiana Lancet.*

### THE ITCHING EYE.

This peculiar condition, most common in childhood, is often the result of uricacidemia, says Dr. John Dunn. He reports the case of a girl of eight who had for three years suffered from an annoying itching of the conjunctiva and lids in spite of more or less constant treatment with various drops and salves. Regulation of her diet and the administration of potassium-iodide in fifteen-grain doses gave complete relief in a few days.—*Va. Med. Semi-Monthly.*

### PAROXYSMAL SNEEZING.

Aside from hay-fever proper, a type of paroxysmal sneezing and running at the nose, occurring at any season of the year in both sexes and all ages, has been observed. Ball states that the most important part of treatment is the removal of obstructions in the nasal passages. Marked amelioration is also afforded by a pill containing a grain of quinine sulphate, one-sixth grain of arsenic iodide, and one-twelfth grain extract of belladonna. Topical applications of cocaine or one per cent. solution of menthol or menthol-camphor also afford marked relief.—*Med. Record.*

---

### GIVING QUININE.

Quinine must not be taken when the bowels are costive, nor when there is diarrhea; nor should it be taken in larger doses than five to eight grains. Always dissolve it with tartaric acid or a mixture of equal parts of dilute phosphoric acid and muriatic acid, using only sufficient to dissolve. For years I have dissolved quinine in glycerine, aiding solution by a little heat: ten drops or minims of pure glycerine will dissolve one grain of quinine; at the same time add a drop or two of the acid mixture.—*Cin. Ec. Med. Journal.*

---

### AN ELEPHANTINE ENEMA.

A little New York girl of three years of age was visiting the country for the first time. Her experience with rubber tubing was limited to the occasional enema of childhood. On the second day of her visit she saw the gardener dragging the sprinkling hose over the lawn. Full of excitement she ran to her mother, exclaiming, "Oh, mamma, what is the man going to do with that injection?"—*The Doctor's Factotum.*

---

### FRICTIONS WITH HOT SALT BEFORE THE DOUCHE.

Cooking salt is mixed with hot water to a semifluid mass with which the limbs and trunk are rubbed for a few seconds before applying the douche, as a hydrotherapeutic measure in cases where a defective reaction is anticipated.—*Semaine Med.*

